# Evaluation of Ultraviolet Type C Radiation in Inactivating Relevant Veterinary Viruses on Experimentally Contaminated Surfaces

**DOI:** 10.3390/pathogens11060686

**Published:** 2022-06-15

**Authors:** Cristina Mendes Peter, Willian Pinto Paim, Mayara Fernanda Maggioli, Rafael Costa Ebling, Kylie Glisson, Tara Donovan, Fernando Vicosa Bauermann

**Affiliations:** 1 Department of Veterinary Pathobiology, College of Veterinary Medicine, Oklahoma State University, Stillwater, OK 74078, USA; cristina.mendes_peter@okstate.edu (C.M.P.); ppaimw@gmail.com (W.P.P.); mayara.maggioli@okstate.edu (M.F.M.); rafaelcostaebling@gmail.com (R.C.E.); 2 Laboratório de Virologia, Departamento de Patologia Clínica Veterinária, Faculdade de Veterinária, Universidade Federal do Rio Grande do Sul, Rio Grande do Sul, Porto Alegre 90040-060, Brazil; 3 Laboratório de Virologia, Departamento de Medicina Veterinária Preventiva, Universidade Federal de Santa Maria, Rio Grande do Sul, Santa Maria 97105-900, Brazil; 4 The Hanor Company of Wisconsin, LLC, Enid, OK 73701, USA; kglisson@hanorusa.com (K.G.); tdonovan@hanorusa.com (T.D.)

**Keywords:** biosecurity, disinfection, swine, UVC, virus

## Abstract

Many swine farms employ UVC treatment in employees’ personal belongings and small tools entering farms as part of the biosecurity protocol to decrease the risk of pathogen introduction into the operation. However, the UVC efficacy in some veterinary viruses is not fully evaluated. This study evaluated the efficacy of ultraviolet type C (UVC) radiation in inactivating seven relevant veterinary viruses: Swine Poxvirus (SwPV), Porcine Reproductive and Respiratory Syndrome Virus (PRRSV), Porcine Epidemic Diarrhea Virus (PEDV), Swine Influenza Virus (SIV), Bovine Viral Diarrhea Virus (BVDV), Porcine Parvovirus (PPV), and Senecavirus A (SVA). The experimentally contaminated materials included polystyrene and filter paper. The samples were exposed to UVC for 5 min (total dose of 360 mJ/cm^2^). The UVC treatment caused a decrease over 4 log10 in SwPV titer on the polystyrene surface, whereas it consistently reduced about 5 log10 in PPV and SVA samples. No viable virus was recovered from PRRSV, PEDV, SIV, and BVDV samples. In filter paper, conversely, the efficacy was reduced. This study provides essential information on the inactivation effectiveness of a specific dose of UVC on important veterinary viruses, further supporting the rational application and strategic guidance for UVC radiation use to disinfect materials.

## 1. Introduction

Controlling the spread of diseases in densely populated swine farms represents a major challenge for the swine sector, requiring strict sanitary measures to support biosecurity and animal health. As part of the biosecurity protocol in many swine farms, UVC radiation chambers are used to treat objects entering farms to decrease the risk of pathogen introduction into the operation [1,2]. The treated objects are typically personnel belongings, including food containers, lunch boxes, cell phones, and small tools. The UVC mechanism of action is based on the formation of thymine, cytosine, or uracil dimers in DNA or RNA. These mutations, consequently, may disable the virus’ ability to replicate [3,4]. The UVC virus inactivation effectiveness of a restricted number of important veterinary viruses has been demonstrated in surfaces, water sanitation, and food processing plants [1,2,5,6,7,8,9,10,11,12]. However, there is a paucity of research on the UVC use and effectiveness on surfaces contaminated with many important swine viruses.

The various methodologies and equipment employed in prior studies hamper comparisons of the UVC treatment efficacy in inactivating different viruses [1,2,5,6,7,8,9,10,11,12]. Furthermore, the UVC radiation dose information is not always available, hindering the determination of effective treatment parameters for the various viruses. It is known that the susceptibility to UV radiation varies among viruses [13]. Despite the knowledge gaps in the efficacy of UVC radiation on specific viruses, the technology is largely used in commercial pig farms [1,2]. Therefore, the objective of this study was to evaluate the UVC’s efficacy in inactivating viruses of clinical importance in swine production on non-porous (polystyrene surface) and porous (filter paper) surfaces that had been experimentally contaminated with seven enveloped and non-enveloped viruses. The study used Swinepox Virus (SwPV), Porcine Reproductive and Respiratory Syndrome Virus (PRRSV), Porcine Epidemic Diarrhea Virus (PEDV), Swine Influenza Virus A (SIV), and Bovine Viral Diarrhea Virus (BVDV)—a primary cattle pathogen belonging to the *Pestivirus* genus, which also includes the Classical Swine Fever Virus (CSFV). Additionally, the non-enveloped virus, Porcine Parvovirus (PPV), and Senecavirus A (SVA) were used.

## 2. Results and Discussion

**Figure 1:**
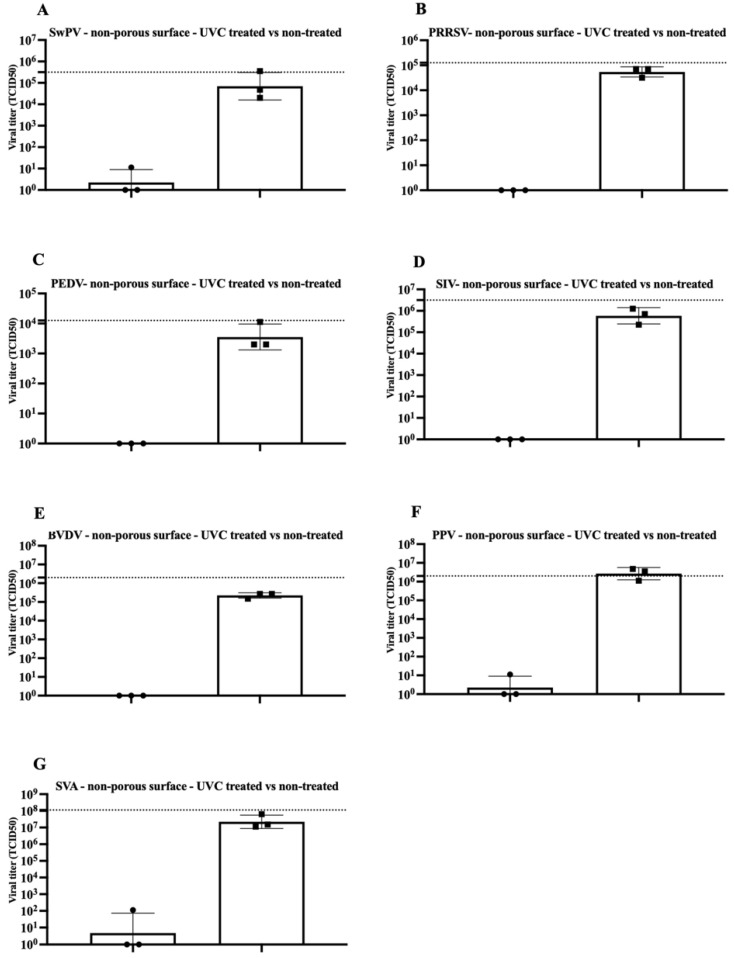
Inactivation of viruses in contaminated non-porous polystyrene surface by UVC treatment. Titration of independent triplicates in UVC treated and non-treated surfaces for Swine Poxvirus (**A**), Porcine Reproductive and Respiratory Syndrome Virus (**B**), Porcine Epidemic Diarrhea Virus (**C**), Swine Influenza Virus (**D**), Bovine Viral Diarrhea Virus (**E**), Porcine Parvovirus (**F**), and Senecavirus A (**G**). Individual result for the triplicate testing is represented by circles (treated group) or squares (control group). Boxes represent the average of the triplicates, and bars represent the standard error of the mean. The dotted line represents the titer of the virus inoculum.

**Figure 2:**
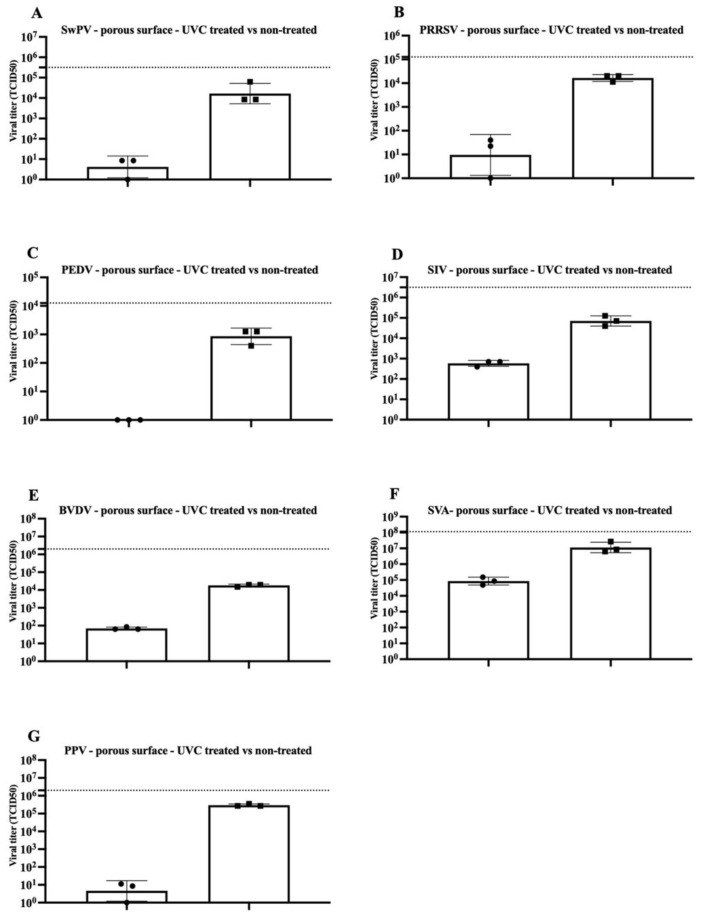
Inactivation of viruses in the contaminated porous surfaces by UVC treatment. Titration of independent triplicates in UVC treated and non-treated surfaces for Swine Poxvirus (**A**), Porcine Reproductive and Respiratory Syndrome Virus (**B**), Porcine Epidemic Diarrhea Virus (**C**), Swine Influenza Virus (**D**), Bovine Viral Diarrhea Virus (**E**), Porcine Parvovirus (**F**), and Senecavirus A (**G**). Individual result for the triplicate testing is represented by circles (treated group) or squares (control group). Boxes represent triplicate averages, and bars represent the standard error of the mean. The dotted line represents the titer of the virus inoculum.

The present study tested the efficacy of a UVC dose of 360 mJ/cm^2^ in inactivating seven viruses on experimentally contaminated surfaces. The results for virus inactivation on the polystyrene (non-porous) surface and the filter paper material (porous surface) are shown in Figure 1 and Figure 2. An infectious virus was not recovered in every replicate in polystyrene-treated surfaces contaminated with SwPV or PPV (one out of the three replicates had measurable viable virus concentration). Conversely, infectious virus was recovered in all but PEDV samples from the treated filter paper material.

After exposure to UVC radiation, there was a consistent decrease in the SwPV titer of about 4 log10 and 3 log10, respectively, in the polystyrene and filter paper material (Figure 1A and Figure 2A). Poxviruses are considered resistant under environmental conditions and are stable over a wide temperature range [14]. The efficacy of UVC on SwPV may suggest that other large, enveloped, double-stranded DNA viruses such as African Swine Fever Virus (ASFV, family *Asfarviridae*) may be susceptible to UVC inactivation. However, specific studies using ASF are required.

The enveloped PRRSV, with titer of a 10^4.75^ TCID_50_, was completely inactivated on a polystyrene surface subjected to UVC treatment. However, when present on a porous surface, the titer reduction was limited to about 3 log10 (Figure 1B and Figure 2B). Whereas the UVC dose used in a previous study evaluating PRRSV inactivation in various surfaces exposed to UVC is not disclosed, a titer reduction of over 10^6^TCID_50_ was obtained using a 10 min treatment [1].

In the current study, no infectious PEDV was recovered from either of the tested surfaces following treatment with a UVC dose of 360 mJ/cm^2^, indicating a titer reduction of at least 3 log10 (Figure 1C and Figure 2C). Similarly, a prior study evaluated the UVC efficacy in PEDV contaminated face masks with a titer of 10^4^ TCID_50_/mL (as a surrogate for SARS-CoV-2). After a 20 to 30 min drying time followed by 10 min UVC treatment, no viable virus was recovered [15].

Following treatment, no infectious SIV was recovered from polystyrene surface samples, compared to a titer of 10^5.7^ TCID_50_ in the control samples. Conversely, in the porous material, the treatment led to partial inactivation with a ~ 2 log 10 titer reduction (Figure 1D and Figure 2D). Another study also found partial inactivation of influenza virus using UVC light. Although, a significant decrease in virus titer was noted in short treatments (less than one minute of UVC exposure). The authors report the treatment successfully reduced the viral titer by approximately 4 log 10 [5].

BVDV virus is a primary cattle pathogen and may also infect pigs [16]. BVDV belongs to the *Pestivirus* genus in the family. BVDV has been used as CSFV surrogate in previous viability studies [17,18,19]. Treatment of polystyrene surface spiked with BVDV resulted in over 5 log10 titer reduction. However, the BVDV titer reduction in porous material was limited to about 2.5 log10 (10^4.5^ TCID_50_ to 10^2^ TCID_50_) (Figure 1E and Figure 2E).

Although non-enveloped viruses, represented in our study by SVA and PPV, were not completely inactivated in polystyrene and porous material, a significant titer reduction for both viruses was observed. A 5 log10 titer reduction was consistently achieved for SVA in the polystyrene surface, whereas at least ~ 2 log10 reduction was reached in paper material (Figure 1F and Figure 2F). Similarly, the effectiveness of UVC in inactivating SVA in three different experimentally contaminated surfaces commonly found in swine farms (cardboard, fabric, and plastic) was evaluated and demonstrated SVA inactivation on plastic surfaces free of organic matter (7 log10 reduction) [2]. However, similar to our findings, UVC treatment efficiency was reduced in porous cardboard samples (2.5-log reduction) [2].

UVC treatment of PPV-contaminated surfaces led to significant titer reduction in both materials, with an approximate 5 log10 for contaminated polystyrene and 4 log10 for contaminated filter material (Figure 1G and Figure 2G). PPV inactivation using UVC was previously evaluated in vaccines, and it was found that treatment with a dose of 13 mJ/cm led to over 5.9 log 10 titer reduction [20].

The study provides information on the effectiveness of a UVC treatment (360 mJ/cm^2^ applied over 5 min) on the inactivation of seven veterinary relevant viruses. Complete inactivation of enveloped viruses in the polystyrene material was noted. However, a viable virus was retrieved from non-enveloped SVA and PPV. Typically, most of the materials treated in farms using UVC are clean personnel belongings such as food containers, cell phones, and small tools. Whereas the materials used in the current study may represent a significant percentage of the surface types exposed to UVC in swine farms, it should be emphasized that the virus inactivation level may vary in the various surface types and materials. It is critical to emphasize that the treatment will likely be significantly less effective on surfaces containing organic matter, which hampers direct UVC light exposure. In addition, the porosity degree or absorbing characteristics significantly impact UVC treatment effectiveness. UVC as a standalone disinfection protocol for surfaces contaminated with organic matter or porous surfaces may increase the risk of virus introduction into farms. Therefore, using additional decontamination steps in these situations would be critical for farm biosecurity.

## 3. Materials and Methods

### 3.1. Viruses and Cells

The cell culture lines to amplify the viruses used in this study are listed in Table 1. The cells were maintained in minimal essential medium (MEM) (Corning^®^, Mediatech, Inc., Manassas, VA, USA) and supplemented with 10% fetal bovine serum (FBS; Seradigma^®^, VWR International, LLC, Radnor, PA, USA), 2 mM L-glutamine (Corning^®^), 1% Antibiotic-Antimycotic 100 X (Gibco^®^, Life Technologies Corporation, Grand Island, NY, USA) and gentamicin (50 μg/mL; Corning^®^). The cell cultures were maintained in an incubator at 37 °C supplemented with 5% CO_2_. The media composition for virus amplification was as described above except for PEDV and SIV amplification. The media used for PEDV and SIV amplification was FBS-free and supplemented with 2 µg/mL of TPCK trypsin (Sigma-Aldrich^®^, St. Louis, MO, USA). The virus stocks were titrated in duplicate using the Reed and Muench method [21]. The virus titers were calculated and expressed in Median Tissue Culture Infectious Dose (TCID_50_) (Table 1).

### 3.2. Study Design

**Figure 3:**
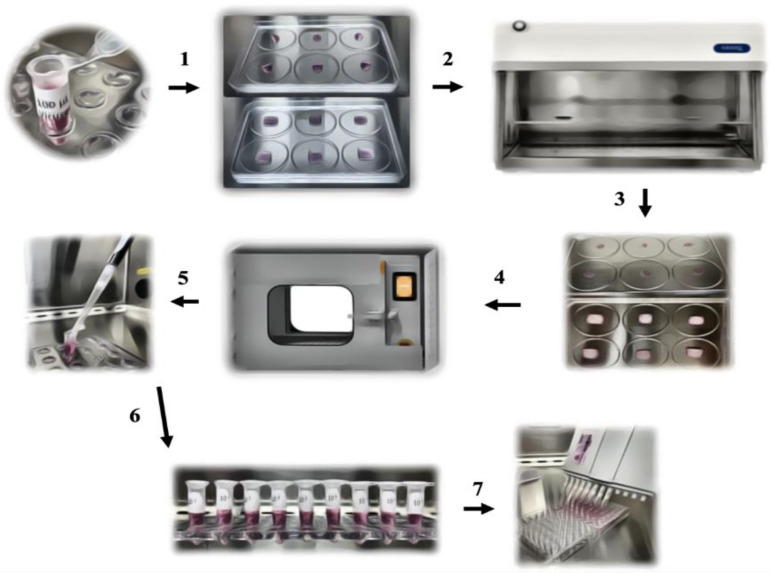
A total of 100 µL of the virus is placed on the testing surface (**1**) and maintained in the biosafety cabinet (**2**), and allowed to dry for 30 min (**3**). Samples were then moved to the UVC chamber for the 5 min treatment (dose of 360 mJ/cm^2^) (**4**). Following treatment, samples were resuspended in 1 mL of MEM (**5**), and eluted samples were serially diluted (**6**) for the virus titration assay (**7**).

The overview of the study design is depicted in Figure 3. To evaluate the UVC inactivation effect on the selected viruses, the commercially available BioShift^®,^ UVC pass-through germicidal chamber (PTC) (Once ™, Plymouth, MN, USA) was used. Four 15 W UVC lamps were present in the chamber. The lamps were distributed in the upper and bottom sides of the units. Two different types of surfaces were evaluated: a polystyrene surface (plastic) and a porous surface (Fisherbrand, filter paper qualitative P8-creped). The UVC radiation dose applied in the samples was monitored using Lutron UVC 254 nm Ultraviolet Light Meter (UVC-254SD) Data Logging. The UVC meter probe was placed adjacent to the tested samples.

Approximately 100 µL of each virus tested was applied to a flat plastic surface or on 1 cm^2^ of the paper filter. The samples were allowed to dry for approximately 30 min in a biosafety cabinet type A2. Dry samples were placed in a biocontainment and moved to the UVC chamber. In the chamber, samples were removed from the biocontainment and placed in the center of the chamber, about 50 cm from the light bulb located in the upper part of the chamber. The samples were then treated for 5 min (treatment time typically used in swine farms). A set of samples was kept UVC-untreated and served as a positive control. After treatment, the samples were resuspended in 1 mL of MEM, and a virus titration assay was conducted.

The UVC-treated and the control samples were titrated using a limiting dilution assay in 96-well plates. Briefly, 50 µL of the viral suspension was added to the wells, with eight replicates of each dilution. Then, 100 µL of the cell suspension was added to all wells (approximately 30 thousand cells/wells). For the PEDV and SIV virus titration assay, plates were prepared 24 h prior to inoculation, and the MEM used for the virus dilution was supplemented with 2 µg/mL of TPCK trypsin (Sigma-Aldrich^®^) and free of FBS. The samples were placed in the incubator at 37 °C, supplemented with 5% CO_2,_ for 72–96 h post-inoculation. The plates were then read in an inverted light microscope. The results were obtained by the identification of the cytopathic effect, and the viral titer was calculated according to the methodology of Reed and Muench [21]. The study was composed of three independent triplicates.

## Figures and Tables

**Table 1:** Characteristics of the viruses used in the study, including the cell line used for virus amplification and the virus stock titer.

Virus	Isolate ID	Viral Family	Envelope	Genetic Material ^1^	Cell Line	Virus Stock Titer
SwPV	NADL	*Poxviridae*	Yes	dsDNA	PK15	10^5.5^ TCID_50_/mL
PRRSV	NADL NA	*Arteriviridae*	Yes	(+)ssRNA	Marc-145	10^5.1^ TCID_50_/mL
PEDV	Colorado 2013	*Coronaviridae*	Yes	(+)ssRNA	Vero	10^4.1^ TCID_50_/mL
SIV	OK-Han1	*Orthomyxoviridae*	Yes	(−)ssRNA	MDCK	10^6.5^ TCID_50_/mL
BVDV	Singer	*Flaviviridae*	Yes	(+)ssRNA	MDBK	10^6.3^ TCID_50_/mL
SVA	HI/2012-NADC40	*Picornaviridae*	No	(+)ssRNA	ST	10^8^ TCID_50_/mL
PPV	Mengeling	*Parvoviridae*	No	ssDNA	ST	10^6.3^ TCID_50_/mL

^1^ Single (ss) or double stranded (ds) nucleic acid viruses were used, including RNA viruses with positive (+) or negative (−) RNA sense.

## Data Availability

Not applicable.
